# Genetic Changes in Experimental Populations of a Hybrid in the *Cryptococcus neoformans* Species Complex

**DOI:** 10.3390/pathogens9010003

**Published:** 2019-12-18

**Authors:** Kelly Dong, Man You, Jianping Xu

**Affiliations:** Department of Biology, McMaster University, Hamilton, Ontario L8S 4K1, Canada; k.dong@mail.utoronto.ca (K.D.); youm1@mcmaster.ca (M.Y.)

**Keywords:** *C. neoformans* × *C. deneoformans*, hybrids, PCR-RFLP, fluconazole resistance, serial transfer

## Abstract

Hybrids between *Cryptococcus neoformans* and *Cryptococcus deneoformans* are commonly found in patients and the environment. However, the genetic stability of these hybrids remains largely unknown. Here, we established mutation accumulation lines of a diploid *C. neoformans* × *C. deneoformans* laboratory hybrid and analyzed the genotypes at 33 markers distributed across all 14 chromosomes. Our analyses found that under standard culture conditions, heterozygosity at most loci was maintained over 800 mitotic generations, with an estimated 6.44 × 10^−5^ loss-of-heterozygosity (LoH) event per mitotic division. However, under fluconazole stress, the observed LoH frequency increased by > 50 folds for the two markers on Chromosome 1, all due to the loss of the fluconazole susceptible allele on this chromosome. Flow cytometry analyses showed that after the 40th transfer (120 days), 19 of the 20 lines maintained the original ploidy level (2N), while one line was between 2N and 3N. The combined flow cytometry, genotyping at 33 markers, and quantitative PCR analyses showed the allelic loss was compensated for by amplification of the resistant *ERG11* allele in eight of the ten fluconazole-stress lines. Our results suggest that hybrids in *C. neoformans* species complex are generally stable but that they can undergo rapid adaptation to environmental stresses through LoH and gene duplication.

## 1. Introduction

The *Cryptococcus neoformans* species complex (CNSC) is an opportunistic human fungal pathogen. It is the cause of the most common fungal infection of the central nervous system and the third most frequent neurological complication in AIDS patients [1]. CNSC contains two evolutionary divergent haploid lineages, *C. neoformans* and *C. deneoformans*, as well as their hybrid, *C. neoformans* × *C. deneoformans*. The *C. neoformans* × *C. deneoformans* hybrids are often aneuploid or diploid, containing alleles from both parental species at most loci [2]. These hybrids have been isolated from both clinical and environmental samples and account for over 30% of the CNSC pathogen population in certain geographic areas [3]. In the laboratory, *C. neoformans* × *C. deneoformans* hybrids can be generated by mating between strains of *C. neoformans* and *C. deneoformans* with opposite mating types (*MAT***a** and *MAT***α**). However, the presence of aneuploid strains in nature suggests that *C. neoformans* × *C. deneoformans* hybrids might not be genetically stable. Indeed, interactions between divergent chromosomes and alleles within the same cell could impact both genome stability and phenotypic expression. However, at present, the stability of the *C. neoformans* × *C. deneoformans* hybrid genome remains largely unknown.

Genome instability may arise from several processes, including genome doubling, gene deletion, gene duplication, chromosomal rearrangements, as well as mitotic crossing-over and gene conversion between alleles on homologous chromosomes in diploid organisms. In diploids, a signature of gene conversion and gene deletion is loss of heterozygosity (LoH). LoH events can be rapid and alter the genome structure of diploid hybrids and serve as a significant form of evolutionary adaptation. LoH has been found in several fungal species, such as *Saccharomyces cerevisiae* [4] and *Candida albicans* [5]. For example, both the common antifungal drug fluconazole and high temperature conditions increased the frequencies of LoH events in *C. albicans*, often involving loss of genes across whole chromosomes [6]. Additionally, LoH has been associated with increased fitness for *Saccharomyces cerevisiae* × *Saccharomyces uvarum* hybrids [7]. In both *S. cerevisiae* and *C. albicans*, the diploid strains often contain highly similar alleles at most loci. Contrarily, in *C. neoformans* × *C. deneoformans* hybrids, there are significant structural and sequence polymorphisms between homologous chromosomes from the two parental species. For example, orthologous genes between these two sister species may exhibit up to 10% nucleotide sequence divergence [8,9]. Such structural polymorphisms and sequence divergence may reduce mitotic crossing-over and influence LoH. In *C. albicans*, fluconazole stress caused a consistent deviation from the normal cell cycle regulation and often resulted in aneuploids [10]. At present, the role of LoH in the adaptation of fluconazole resistance has yet to be elucidated in *C. neoformans* × *C. deneoformans* hybrids.

**Figure 1:**
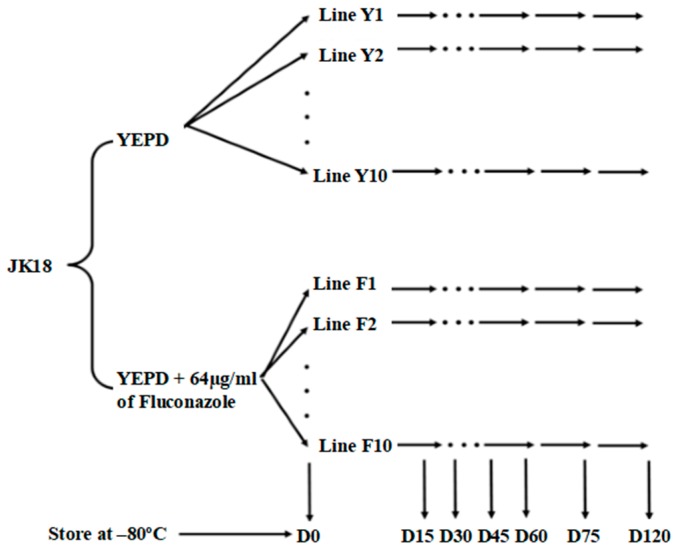
Representation of the mutation accumulation lines. Ten lines of JK18 were plated on YEPD agar, and YEPD agar supplemented with 64 µg/mL of fluconazole. Each of the 20 independent lines were transferred every three days for a total of 40 transfers over 120 days. Cells were periodically stored on days 15, 30, 45, 60, 75, and 120.

The objective of this study is to investigate the stability of a *C. neoformans* × *C. deneoformans* hybrid and the effect of the antifungal drug fluconazole stress on LoH. Strain JK18 was selected for this experiment. JK18 was created by mating two strains, a clinical *C. neoformans* isolate CDC15 (serotype A, *MAT***α**) and a model *C. deneoformans* lab strain JEC20 (serotype D, *MAT***a**) and it was heterozygous at all 73 PCR-RFLP markers [11]. Strain CDC15 has a fluconazole minimum inhibitory concentration (MIC) of 64 μg/mL in liquid medium, while strain JEC20 has a fluconazole MIC of 4 μg/mL. JK18 also has a fluconazole MIC of 64 µg/mL. Vogan et al. revealed that the high fluconazole resistance of strain CDC15, as measured on solid medium, was controlled by three quantitative trait locus (QTL) regions, two located on Chromosome 1 and one located on Chromosome 3 [11]. The two regions around markers CNA00290 and CNA07470 on Chromosome 1 contain the fluconazole target gene *ERG11* and *AFR1*, a major transporter for triazoles in *C. neoformans* [11,12]. The region closely linked to marker CNA00290 on Chromosome 1 contributed up to 36% of the total MIC variance among progeny from the CDC15 × JEC20 cross. Specifically, at all three marker loci, strains that are homozygous for the CDC15 (A) allele or heterozygous with both CDC15 (A) and JEC20 (D) alleles had higher MICs than those that are homozygous for the JEC20 (D) allele. Here in this study, we used mutation accumulation (MA) approach (Figure 1) and the hybrid strain JK18 as the starting culture to investigate its genetic stability and how fluconazole stress could impact its genetic change.

## 2. Results

**Figure 2:**
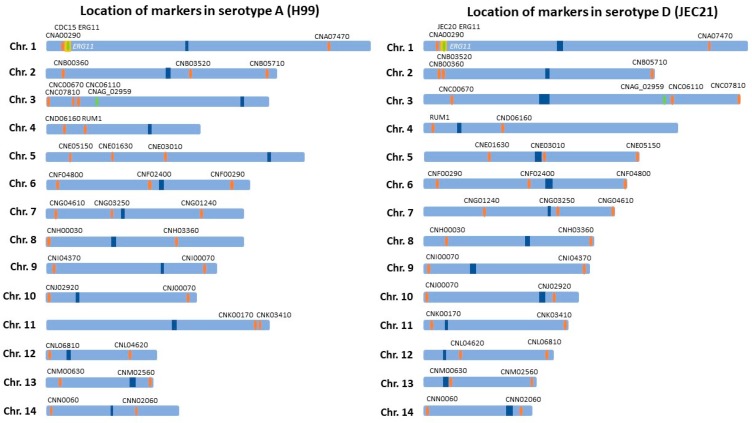
Chromosomal locations of the 33 PCR-RFLP markers used for assaying genotypes in this study. Shown here are the respective positions of the 33 PCR-RFLP markers in two strains H99 and JEC21 representing the two sequenced and annotated genomes serotype A and serotype D genomes, respectively. Markers CNA00290 and CNA07470 on chromosome 1 flank the fluconazole target gene *ERG11* while marker CNN02060 on chromosome 14 is located close to a fluconazole QTL. CNAG_02959 is the marker used as an endogenous reference control for qPCR and is located on Chromosome 3. Dark blue indicates the location of the centromeres.

In this study, strain JK18 was used to establish twenty MA lines, with ten of the lines grown on normal rich medium YEPD (these are called the Y-series lines) and the other ten lines on YEPD supplemented with the antifungal drug fluconazole (these are called the F-series lines). At the end of forty transfers (after 120 days), the cultures were assayed for susceptibility to fluconazole and genotyped at 33 molecular markers with two marker loci on each of the 14 chromosomes (Figure 2). The 33 markers were chosen based on their distributions among chromosomes and their associations with QTLs related to several phenotypic traits such as fluconazole MIC, the productions of melanin and capsules, cell size, and cell wall thickness, as demonstrated previously. Below we describe the genotypic and fluconazole susceptibility changes of the experimentally evolved cultures.

### 2.1. Patterns of Heterozygosity of the Experimentally Evolved Lines

Among the 10 Y-series lines, three (Y1, Y7, and Y8 lines) were completely heterozygous at all 33 marker loci (Table 1). The remaining seven lines lost heterozygosity at either one locus (Y3 and Y5 lines), two loci (Y4 and Y9 lines), three loci (Y2 and Y6 lines), and five loci (Y10 line). In total, of the total 330 combinations of Y lines (10) X loci (33), 17 lost heterozygosity (Table 1), corresponding to a frequency of 5.2% over the 800 mitotic generations, or 6.44 × 10^−5^ LoH event per mitotic division. Those that lost heterozygosity involved 13 marker loci distributed on seven chromosomes (Table 1). Of these 17 LoH events, seven resulted in homozygosity for the JEC20 (D) alleles, while the remaining ten were homozygous for the CDC15 (A) alleles.

Among the 10 F-series lines, none were completely heterozygous at all 33 marker loci by the end of the 120 days (Table 1). Nine of the ten lines lost heterozygosity for both marker loci on Chromosome 1, and all resulted in homozygosity for the CDC15 (A) alleles. Only line F2 maintained heterozygosity for the two marker loci on Chromosome 1. Excluding markers on Chromosome 1, there were 12 other LoH events involving 9 markers on six chromosomes in the 10 F-series lines. Of these 12 LoH events, four resulted in homozygosity for the JEC20 (D) alleles, while the remaining eight resulted in homozygosity for the CDC15 (A) alleles, a pattern similar to that of the 10 Y-series lines. The overall LoH frequency among the F-series lines was 9.1% (30 LoH events in 330 combinations of F lines × loci) after 800 mitotic divisions. This frequency is just under twice of that (5.2%) under the non-fluconazole growth condition. However, excluding marker loci on Chromosome 1, the LoH frequency in the F-series lines was 4.5%, similar to that of the Y-series lines. Interestingly, under both experimental conditions, marker loci on Chromosomes 4, 6, 10, and 11 maintained their heterozygosity in all 20 lines while those on Chromosomes 12, 13, and 14 showed LoH in selected lines of both treatment groups.

Three QTL were identified for fluconazole MIC in broth, and three for MIC on solid medium by Vogan et al. [11]. These included regions around markers CNA00290 and CNA07470 on Chromosome 1; CNC06110 on Chromosome 3; CNE01630 on Chromosome 4; and CNN02060 on Chromosome 14, with CNA00290 contributing to both media conditions. Here we screened the genotypes at the five loci for all MA lines. No LoH event was observed for marker CNC06110 (Chr3) nor marker CNE01630 (Chr4) in either treatment group. For marker CNN02060 (Chr14), one Y-series line (Y2) lost the JEC20 (D) allele while three F-series lines (F2, F5, and F6) lost the JEC20 (D) allele. In contrast to the overall similarity of the three markers between the two treatment groups, the two marker loci on Chromosome 1 (CNA00290 and CNA07470) showed a significant difference in LoH events between the F-series and the Y-series treatment groups (*p* = 0.0005). Specifically, at marker loci CNA00290 and CNA07470, only one of the ten Y-series lines (line Y10) became homozygous for the CDC15 (A) alleles. In contrast, nine of the ten F-series lines became homozygous for the CDC15 alleles for both markers on Chromosome 1 (Table 1). The only exception was line F2, which remained heterozygous for both marker loci on Chromosome 1. Interestingly, line F2 was homozygous with the CDC15 (A) alleles at loci CNL06810 (Chr12), CNN0060 (Chr14), and CNN02060 (Chr14).

As shown in Table 1, of the total 47 LoH events for the 20 mutation accumulation lines, 32 involved marker loci on two arms of the same chromosomes. These included three chromosomes in the Y-series lines and 13 pairs in the F-series lines. They were located on four of the 14 chromosomes (i.e., Chromosomes 1, 9, 13, and 14). Since the markers on these four chromosomes were located far apart from each other, these LoH events likely represented whole chromosomal losses. The remaining 15 LoH events involved only one of the marker loci on each chromosome. These 15 events likely included chromosomal breakage, mitotic crossing-over, or gene conversion. Twelve marker loci were involved in the 15 LoH events and these 12 loci were located on seven chromosomes, Chromosomes 2, 3, 5, 7, 8, 12, and 14 (Table 1). 

### 2.2. Genotype on Chromosome 1 in the F-series Lines

Because of the high frequency of LoH for the two marker loci on Chromosome 1 in the fluconazole treatment group, we further investigated the approximate time frames that LoH events occurred at these two marker loci in these lines. Genomic DNA from stored cultures at Days 15, 30, 45, 60, and 75 were all extracted from the nine F-series lines and one Y-series line (Y10) where LoH events for Chromosome 1 were observed at Day 120 cultures. Table 2 shows the results of our analyses for marker loci CNA00290 and CNA07470 on Chromosome 1. By Day 15 (i.e., the fifth transfer), five of the ten F-series lines (lines F1, F3, F4, F7, and F10) had lost the JEC20 (D) alleles for both marker loci on Chromosome 1. By Day 30 (10th transfer), lines F8 and F9 also lost the JEC20 (D) alleles (Table 2). By Day 45, lines F5 and Y10 lost the JEC20 alleles. Line F6 lost the JEC20 (D) alleles by Day 60, and only line F2 remained heterozygous after Day 60 and maintained heterozygosity until the end, at Day 120 (Table 2). 

Based on these results, the LoH frequency for the two marker loci on Chromosome 1 under fluconazole stress over the 120 days was 90%. The minimum average number of mitotic divisions that the 10 F-series lines went through before losing heterozygosity (or had not lost heterozygosity at the end for line F2) was around 240 mitotic divisions [(100+100+100+100+100+200+200+300+400+800)/10]. Thus, the observed rate of LoH for marker loci on Chromosome 1 under 64 µg/mL fluconazole stress for strain JK18 was 3.75 × 10^−3^ per mitotic division. This rate was over 50 times that observed for the Y-series lines and other marker loci in the F-series lines.

### 2.3. Fluconazole MIC of the Mutation Accumulation Lines

Following the Clinical and Laboratory Standards Institute (CLSI) protocol, we determined the fluconazole MICs of the end cultures for all 20 MA lines (i.e., after 40 transfers at 120 days). The results are shown in Table 1. Of the 10 Y-series lines, all but one line (Y10) had reduced fluconazole MIC. However, the reductions were relatively minor, between 2–4 folds, within the acceptable range of experimental error under the CLSI guideline. In contrast, line Y10 had increased fluconazole MIC (128 µg/mL) compared to the original clone of JK18. Interestingly, line Y10 lost the JEC20 (D) alleles for both marker loci on Chromosome 1 while maintaining the CDC15 (A) alleles (Table 1). In comparison to the MIC values shown by the Y-series lines, all lines of the F-series showed increased MICs over the initial clone of JK18 (Table 1). This included line F2 that was heterozygous containing both the JEC20 (D) and CDC15 (A) alleles at the two marker loci on Chromosome 1. 

### 2.4. Colony Sizes of the Experimentally Evolved Cultures on YEPD Media Supplemented with Various Concentrations of Fluconazole

**Figure 3:**
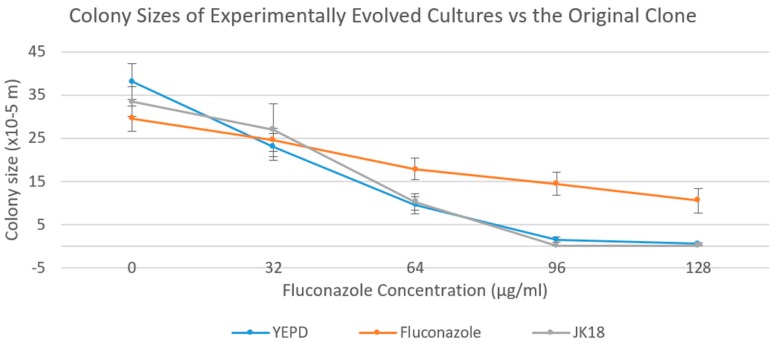
Mean colony size for the mutation accumulation lines at 120 days after serial transfer on YEPD agar (YEPD or Y-lines) and YEPD agar + 64 µg/mL of fluconazole (Fluconazole or F-lines) over various concentrations of fluconazole. The original clone of JK18 was included for comparison. Based on a one-way ANOVA, there was statistically significant difference (*p* < 0.05) between the Y-lines and F-lines at 0, 64, 96, and 128 μg/mL. There was no significant difference between the Y-lines and JK18 at any concentration of fluconazole.

Xu et al. determined that MIC of fluconazole can be determined by measuring colony size as a method of quantification [13]. Since the mutation accumulation lines were maintained on solid agar media, we further investigated the growth of the evolved cultures on agar medium supplemented with various concentrations of fluconazole. Here, colony size was used as a proxy for vegetative growth rate on solid medium, with a bigger colony reflecting more growth. Figure 3 shows the mean colony sizes of the evolved lines after 40 transfers at five fluconazole concentrations (0, 32, 64, 96, and 128 µg/mL). Our results demonstrated that, on average, the Y-series lines were similar to the original clone of JK18 in the growth pattern, starting with a relatively large colony size of around 37 × 10^−5^ m in diameter at 0 µg/mL of fluconazole before rapidly decreasing with increasing fluconazole concentration. At 96 µg/mL of fluconazole, there was almost no growth for most of the evolved clones (except the Y10 line), with the mean colony size approaching zero. In comparison, the colony sizes for cultures evolved under fluconazole stress were initially smaller, with a mean diameter around 29 × 10^−5^ m. However, the decrease in colony size of the F-series cultures at increasing fluconazole concentrations was smaller than those of the Y-series cultures, with noticeable growth at 128 µg/mL of fluconazole for all the F-series lines (Figure 3). Supplementary Figure S1A,B show the colony sizes of individual Y-series lines and F-series lines, respectively. As shown in Figure S1, the growth pattern of line Y10 was similar to those of the F-series lines.

### 2.5. Genotypic Contribution to MIC

**Figure 4:**
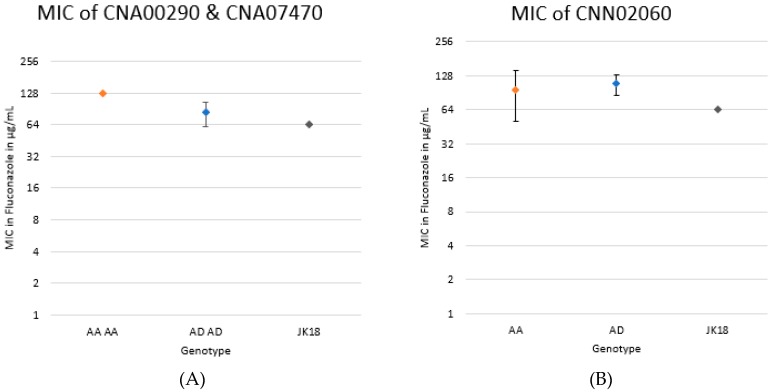
Relationships between fluconazole MIC and genotypes at two loci on Chromosome 1 (Figure 4A) and one locus on Chromosome 14 for cultures on Day 120 (Figure 4B). The MIC for each genotype was determined by ten replicates each for genotypes AD and A compared to the original strain JK18. This was determined as the minimal concentration of fluconazole on which there were no colonies observed. Based on an unpaired t-test, there was a significant difference between A and AD in the two loci on Chromosome 1 (*p* < 0.0001) but no significant difference between A and AD in CNN02050 (*p* = 0.4527). All replicates with genotype A in CNA00290 and CNA07470 had the same MIC.

Upon examination of the marker loci CNA00290 and CNA07470 on Chromosome 1, those that were homozygous for the CDC15 (A) allele had a significantly higher MIC than either those that were heterozygous at the two loci or the original clone JK18 (*p* < 0.0001). As expected, there was no significant difference between MICs of those that were heterozygous at the two loci and JK18 (which was also heterozygous at these two marker loci; Figure 4A). Furthermore, there was no significant difference in either fluconazole MIC or colony size between those that were heterozygous with both the CDC15 (A) and JEC20 (D) alleles and those that were homozygous for the CDC15 (A) allele in the other QTL region CNN02060 located on Chromosome 14 (Figure 4B). 

### 2.6. Copy Number of ERG11 in the Experimentally Evolved Cultures

**Figure 5:**
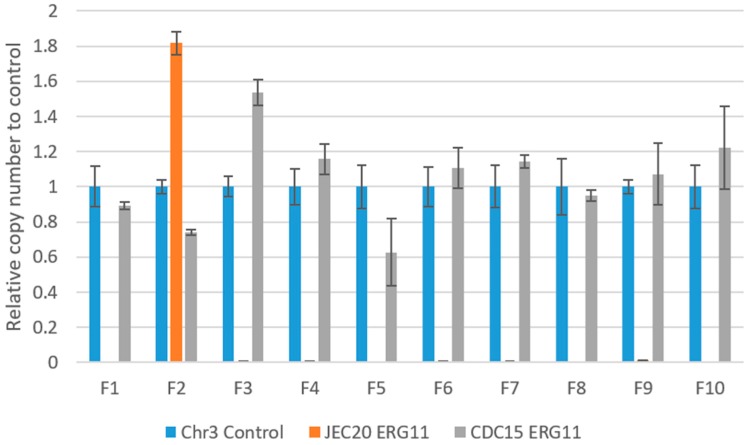
qPCR quantification of the *ERG11* gene in fluconazole treated mutation accumulation lines. A single copy gene on Chromosome 3 was used as the reference control, and all copy numbers were normalized to the Chr3 control. The Chr3 control is present in two copies in JK18, as it contains both the JEC20 and CDC15 alleles. Only the Day120 cultures were analyzed.

*ERG11* is located on Chromosome 1, which is typically present in a single copy in the genome in haploid strains of *C. neoformans* and *C. deneoformans*. It encodes for cytochrome P450 lanosterol-14-α-demethylase, which is the target for fluconazole [14]. However, *ERG11* has been previously found to be often amplified under fluconazole stress [15]. Here we investigated the copy number of the JEC20 and CDC15 alleles at the *ERG11* locus in the mutation accumulation lines under fluconazole stress after the final (i.e., 40th) transfers. Figure 5 shows the relative copy number of the *ERG11* gene from both the JEC20 and CDC15 parents. The copy numbers were all normalized to the reference marker CNAG_02959 on Chr3. Marker CNAG_02959 was chosen as our qPCR reference here based on the results by Sionov et al. [12]. In that study, Chromosome 3 (and marker CNAG_02959 on this chromosome) was stable under various fluconazole selection pressure. Specifically, CNAG_02959 maintained its single-copy status in all the strains derived from the haploid strain H99 under various fluconazole concentrations. In contrast, other chromosomes such as Chromosomes 1, 4, 10, and 14 showed differences in copy numbers among the H99 derivatives exhibiting various levels of fluconazole heteroresistance. Of the ten F-series lines in our study, seven had two copies of the CDC15 (A) allele of *ERG11* gene and one (line F3) had three copies of the CDC15 *ERG11* allele. The only F-series line with only one copy of the CDC15 *ERG11* gene was line F5. Interestingly, in line F2 where heterozygosity for Chromosome 1 was maintained for both markers, there seemed to be about four copies of the JEC20 *ERG11* allele and close to two copies of the CDC15 *ERG11* allele (Figure 5). Overall, the results suggested that in most cases, fluconazole stress was associated with loss of the JEC20 (D) allele and amplification of the CDC15 (A) allele. However, amplification of both the JEC20 and/or the CDC15 allele in line F2 might also contribute to its high fluconazole MIC.

### 2.7. Ploidy Level

**Figure 6:**
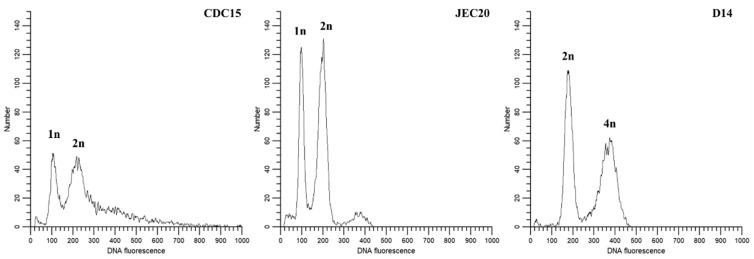

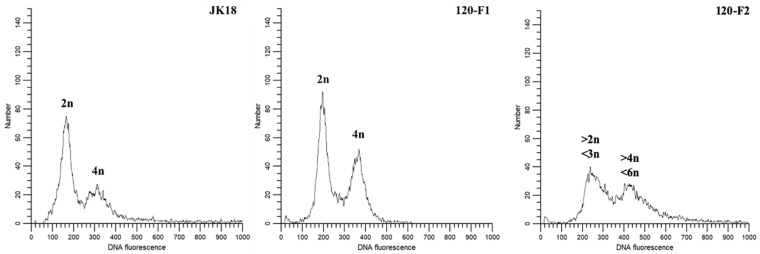
Representative profiles of fluorescence-activated cell sorting (FACS) analyses. Strains CDC15 and JEC20 are haploid reference strains and parents of JK18. D14 is a diploid reference. 120-F1 and 120-F2 are representative profiles of the derived mutation accumulation line cultures at day 120.

All the Day120 cultures of the 20 mutation accumulation lines were tested for genome content by fluorescence-activated cell sorting (FACS). The results demonstrated that 19 of the 20 lines maintained their ploidy levels at around 2N, similar to the original clone of JK18. The exception was line F2, which showed a ploidy level greater than 2N and close to 3N (Figure 6). Figure 6 shows the FACS profiles of three reference strains as well as the founder strain JK18 and two representative Day 120 cultures from lines F1 and F2. However, due to the coarse nature of FACS, minor gains or losses of genomic DNA contents including the gain and loss of entire chromosomes could not be detected or confirmed by FACS profile alone. 

## 3. Discussion

In this study, we experimentally evolved a diploid *C. neoformans* × *C. deneoformans* laboratory hybrid strain JK18 through serial transfers to investigate its genome stability during vegetative growths and how fluconazole stress could impact genome stability. Our serial transfers maximized genetic drift at each round of sub-culturing. Strain JK18 was predetermined to be heterozygous at all 73 marker loci analyzed previously [11], with each locus containing alleles from both parental strains JEC20 and CDC15. The stability of the evolved cultures was examined by LoH events over 33 marker loci, with at least one marker locus distributed on each arm of the 14 non-homologous chromosomes in the genome. Our analyses showed that LoH occurred at a minimal rate of 6.44 × 10^−5^ per mitotic division. However, under 64µg/mL fluconazole stress, the observed rate of LoH for markers on Chromosome 1 for strain JK18 was 3.75 × 10^−3^ per mitotic division, over 50 times higher than the observed baseline rate for other marker loci. Our results, based on the patterns of LoH, qPCR, and FACS, suggest that several types of genetic changes have likely happened during the mutation accumulation of this *C. neoformans* × *C. deneoformans* laboratory hybrid. These changes included whole or partial chromosome loss, mitotic crossing-over or gene conversion, and whole or partial chromosomal duplication. Below we discuss the potential mechanisms for the observed results and their implications.

### 3.1. Relationship Between Genetic Changes in Chromosome 1 and Susceptibility to Fluconazole

Overall, we only observed a much higher rate of LoH on Chromosome 1 in the F-series lines than in the Y-series lines. The increased rate was due to LoH events involving two marker loci CNA00290 and CNA07470 on Chromosome 1 under fluconazole stress. In a previous study, it was demonstrated that genomic regions around these two marker loci explained a significant percentage of the phenotypic variance in MIC for progenies derived from the JEC20 and CDC15 cross, with regions around CNA00290 explaining 36% of the colony size variation on solid agar media and 14% of the MIC variation in liquid broth [11]. The other marker locus on Chromosome 1, CNA07470, explained 11% of the MIC variation in broth but none on solid agar media [11]. In these two regions, there was a significant difference in LoH rate between the two treatment groups (*p* = 0.0005). Our result contrasts with previous work in *C. albicans,* where the exposure to fluconazole increased the proportion of whole-genome LoH [16]. Here, we observed a focused LoH on Chromosome 1 rather than a whole-genome instability in response to fluconazole as concluded by Forche et al. and Harrison et al. in *C. albicans* [6,10]. This may due to the differences in the original ancestral strain, as *C. albicans* strains are typically diploid while *C. neoformans* strains are typically haploid. On the other hand, markers around two other QTL regions, CNC06110 on Chromosome 3 and CNN02060 on Chromosome 14, showed no difference in either LoH or genotype frequencies between the two mutation accumulation groups. Genomic regions around the two marker loci CNC06110 and CNN02060 explained about 4% of the fluconazole MIC variation among progeny from the hybrid cross between strains JEC20 and CDC15 [11].

The two regions around markers CNA00290 and CNA07470 on Chromosome 1 contain the fluconazole target gene *ERG11* and *AFR1*, a major transporter for triazoles in *C. neoformans* [11,12]. Marker locus CNA00290 is about 7kb from *ERG11*. As demonstrated in a previous study, progeny homozygous for the CDC15 (A) alleles at marker loci CNA00290 and CNA07470 were more resistant to fluconazole than those that were homozygous for the JEC20 (D) alleles or heterozygous containing both the JEC20 (D) and CDC15 (A) alleles [11]. In addition, Sionov et al. showed that increased copy number of these two genes through chromosomal duplication was associated with increased fluconazole resistance in a haploid strain of *C. neoformans* H99 [12,15]. Our results are consistent with the major contributions of genes around these two marker loci to fluconazole resistance. Furthermore, although there was no LoH at either marker loci on Chromosome 1 for line F2, there was evidence for increased copy numbers for both the CDC15 (A) and JEC20 (D) alleles at the *ERG11* locus in this line.

In this study, we used CNAG_02959 as an internal reference for qPCR in assaying the copy number of *ERG11* gene for the F-series strains after 120 days of mutation accumulation. However, while the choice of CNAG_02959 was originally based on its stability under fluconazole stress in a haploid strain H99 [12], our inferences of *ERG11* gene copy number were based on two additional pieces of evidence as well. Specifically, our FACS analyses showed that nine of the ten F-series strains (except F2) in our study were consistent with diploidy, similar to the starting clone of JK18 and the diploid reference strain D14. Second, in our diploid hybrid JK18 and all the 10 F-series derivatives at day 120, all three marker loci on Chromosome 3 maintained their heterozygosity, containing alleles from both the serotype A and D parents (Table 1), consistent with Chromosome 3 being heterozygous and disomic in these strains. Taken together, we believe that there are two copies of CNAG_02959 in nine of the ten evolved strains (except line F-2) and that our inferences of the copy number of the JEC20 and CDC15 *ERG11* alleles based on the copy number of CNAG_02959 in the nine F-series strains are robust. The only strain we are unsure of the copy number of CNAG_02959 is line F2. However, based on the observed heterozygosity and FACS result, strain F2 likely contains two copies of most chromosomes, including Chromosome 3 (and the gene CNAG_02959 on Chromosome 3). The only exception might be Chromosome 1. For Chromosome 1, based on qPCR result of *ERG11*, there are likely close to two copies of *ERG11* from CDC15 and about four copies from JEC20. If *ERG11* gene copy numbers were representative of chromosome 1 copy numbers, and given Chromosome 1 is about 2.3 million base pairs in length, the extra copies of Chromosome 1 would contribute over 9 mb extra to the genome size, leading to the FACS profile similar to that observed in Figure 6 for strain F2 (with a genome content between 2N and 3N). However, other possibilities could also explain the observations, including partial duplications of Chromosome 1 and/or whole or partial duplications of other chromosomes. Additional analyses are needed in order to understand the genome structure of strain F2. 

In addition, we cannot exclude the possibility that other genetic changes, including novel mutations accumulated during mutation accumulation, could have contributed to the observed changes in fluconazole susceptibility among the F-series lines. As shown previously [12,15,17], further analyses such as high-coverage whole genome sequencing, comparative genome hybridization, and/or qPCR analyses based on more genes distributed throughout the genome could help reveal more detailed information about other potential genetic changes (including copy number variations of other genes, chromosomal segments, whole chromosomes, and nucleotide substitutions) among these evolved strains.

### 3.2. Biased Loss of Alleles on Other Chromosomes

Aside from the biased LoH in favor of maintaining the CDC15 (A) alleles on Chromosome 1 in the F-series lines, there seemed a slight but statistically insignificant preference for the CDC15 (A) alleles during LoH in other parts of the genome. Previous studies have shown that *C. neoformans* strains were more prevalent in clinics and showed an overall higher virulence, including being capable of growing at a higher temperature than *C. deneoformans* strains [11,18]. Thus, the preference for maintaining the *C. neoformans* (CDC15, A) alleles over *C. deneoformans* (JEC20, D) alleles after LoH events may be in part due to the relatively high-temperature environment (37 °C) used during our experimental evolution, where cells with only the CDC15 (A) alleles after LoH events likely grew faster than those with JEC20 (D) alleles. It has been found that the loss or maintenance of which parental haplotype is associated with the temperature preference of the parents as well we the temperature that the hybrid evolved in *S. cerevisiae* × *S. uvarum* hybrids [19]. High temperature has also been found to increase LoH events in *C. albicans* [16]. However, certain responses could only be observed in a low temperature environment, such as the preferential retention of the cryotolerant allele in *S. cerevisiae* × *S. uvarum* hybrids [19]. Experimental evolution at different temperatures is needed to investigate the effects of temperature on LoH and thermo-adaptation in *Cryptococcus* hybrids. 

Based on the observed genetic changes among the mutation accumulation lines, the increased rate of LoH events in fluconazole treatment was only observed for the two marker loci on Chromosome 1. We believe that the selection for homozygosity of the CDC15 (A) alleles, not increased mutation pressure by fluconazole stress, was likely the driver for the observed increase in LoH among the F-series lines. Besides, CNA00290 and CNA07470, which are around 1159 kb apart, have the same genotype for all 20 mutation accumulation lines. Our results suggest that when LoH events happened to the far apart loci on one chromosome, a whole chromosome was likely lost. The marker loci CNA00290 and CNA07470 flank ERG11, the primary gene implicated in fluconazole resistance. This result implies that LoH of *ERG11* selecting for the A (CDC15) allele can overcome drift under fluconazole stress. Additional markers covering more regions of Chromosome 1 are needed in order to confirm the hypothesis. Whole chromosome loss could happen through mis-segregation during mitoses, similar to the genetic alterations that have been hypothesized to drive tumorigenesis in animals [20].

### 3.3. Implications in Evolutionary Biology and Clinical Practice

Interestingly, we observed a decrease in colony size on YEPD agar media without fluconazole for the F-series of cultures. This result suggests that there may be tradeoffs between growth in the presence and absence of fluconazole in experimental populations of the *C. neoformans* × *C. deneoformans* hybrids. Specifically, as expected, the cultures continuously exposed to fluconazole showed increased growth ability in the presence of fluconazole, likely due to the accumulation of adaptive genetic changes for the specific environment. However, in the absence of fluconazole, these fluconazole-adapted cultures did not perform as well as those not adapted to an environment with fluconazole.

At present, whether a similar tradeoff existing in clinical strains of *C. neoformans* remains unknown. If there were tradeoffs, we should expect a negative correlation between fluconazole MIC and vegetative growth in the absence of fluconazole. However, it is also known that prolonged exposure to stress could select for compensatory mutations to overcome the potentially initial costs associated with mutations to stress resistance. Understanding the potential tradeoffs of drug resistance and its biological mechanisms has significant practical implications. For example, there are growing concerns over the increase in resistance of fungal pathogens to antifungal agents, including fluconazole. One of the major topics of discussion at both the global and local levels is how to prevent and reduce drug resistance in patients. We need to develop management protocols that not only minimize the origin of resistance mutations but also reduce their compensatory mutations.

## 4. Materials and Methods

### 4.1. Strains

Four strains were used in this study: JEC20 (*C. deneoformans*, *MAT***a**, D), CDC15 (*C. neoformans*, *MAT***α**, A), JK18 (*C. neoformans* × *C. deneoformans*), and D14. JK18 is a mating product between strains JEC20 and CDC15 and has shown to be heterozygous at most of the Polymerase Chain Reaction-Restriction Length Polymorphism (PCR-RFLP) markers examined in previous studies [11,21]. JK18 has a fluconazole MIC of 64 µg/mL. Strain D14 was a diploid fusion product of *C. deneoformans* [22]. Strains JEC20, CDC15, and D14 were used as references for identifying the ploidy of experimentally evolved cultures.

### 4.2. Mutation Accumulation Lines

To establish experimental evolution lines, a stock culture of strain JK18 from −80 °C freezer was first streaked onto the rich yeast extract-peptone-dextrose (YEPD) agar and incubated at 37 °C for two days. A single colony from the plate was then used to streak for ten independent experimental evolution lines on YEPD agar and another ten independent lines on YEPD agar + 64 µg/mL of fluconazole. The plates were incubated at 37 °C for three days. By the end of the third day, the cells increase from 1 cell to approximately ~10^6^ cells, accounting for about 20 mitotic divisions. Every three days, the transfer within each line was made by streaking a random colony to a new plate, and the plates were incubated at 37 °C. The experimental evolution was completed over 120 days. Cells on days 15, 30, 45, 60, 75, and 120 were collected and stored in 20% glycerol in −80 °C freezer. These cells corresponded to approximately 100, 200, 300, 400, 500, and 800 mitotic divisions, respectively, after the initial setup (see Figure 1). 

### 4.3. Genotyping

At the end of the experimental evolution phase, the stored cells from each time point were all plated on YEPD and allowed to grow at 23 °C for three days before they were harvested for DNA extraction. Genomic DNA was extracted from the original culture of JK18, as well as the 20 evolved cultures. These DNA samples were extracted then used as templates for genotyping using PCR-RFLP markers as described previously by Vogan et al. [23]. A total of 33 PCR-RFLP markers that were heterozygous in strain JK18 were selected and genotyped for each evolved culture, with at least two markers on each chromosome and covering both chromosome arms in the reference genome of strain JEC21 (an isogenic strain of JEC20) (Figure 2); [24]. These included all five markers previously identified as contributing quantitative trait loci to fluconazole MIC (CNA00290, CNE01630, CNN02060, CNA07470, and CNC06110 [11]. The specific marker loci that were analyzed are shown in Table 1. The primers, PCR parameters, restriction enzyme digests, and gel electrophoresis all followed those described previously in Vogan et al. [11,23].

### 4.4. Minimum Inhibitory Concentration for Fluconazole

The minimal inhibitory concentration (MIC) of fluconazole for each culture was assayed both in liquid medium and on solid agar. MIC in liquid medium was obtained following the CLSI microbroth dilution protocol. For colony growth and MIC on solid agar, we followed the method described in Xu et al. [13]. Specifically, we tested the effects of fluconazole on the colony size on solid YEPD media supplemented with fluconazole concentrations of 0, 32, 64, 96, and 128 µg/mL. For each culture, cryptococcal cells were picked and suspended in 400 µL of distilled water before being streaked for single colonies onto YEPD plates at the respective fluconazole concentrations. The cells were left to grow in a 30 °C incubator for two days before the diameter of colonies was measured under a microscope. For each culture, 10 colonies were measured at each fluconazole concentration. The MIC for growth on solid medium was determined as the lowest concentration of fluconazole where no colonies were observed [13]. If there was growth on agar medium containing 128 µg/mL of fluconazole, the MIC was noted to be > 128 µg/mL. The colony sizes were assessed at each fluconazole concentration level by performing one-way ANOVA. The differences in MIC between genotype A and AD at each locus was determined using unpaired T-tests.

### 4.5. qPCR Copy Number Variation of the ERG11 Gene

In addition to genotyping for evidence of LoH, we also used qPCR to determine the copy number of the *erg11* gene for the cultures. Here, the JEC20 and CDC15 specific primers were designed based on the published genome sequences of representative strains of *C. deneoformans* (JEC21) and *C. neoformans* (H99) using the program *AlleleID*. The primers for the JEC20-specific *ERG11* gene were (5′–3′) gagtcccttcagtcttatc (forward) and catggatttgaggaggtc (reverse); those for the CDC15-specific primers were (5′–3′) ccatgctcattggattcc (forward) and gggaatgtagtgaaagaca (reverse). Prior to qPCR, all extracted DNA samples were standardized to 5 ng/mL for consistency using the NanoDrop. Following the recommendation of Sionov et al. [12], the Chromosome 3 gene CNAG_02959 was used as an endogenous reference control of the single-copy gene. The primers for amplifying fragments of CNAG_02959 from both the JEC20 and CDC15 genomes were (5′–3′) gaagatggcgagcctgaca (forward) and cacttcgagccttcttcttcatg (reverse). All strains were run with three replicates and analyzed using CNAG_02959 as a standard. 

### 4.6. Ploidy Analyses

The ploidy of JK18 and its experimentally evolved derivatives were determined by fluorescence-activated cell sorting (FACS), following the protocol described in Skosireva et al. [25]. Briefly, cells were harvested from overnight YEPD liquid medium by centrifugation, washed in phosphate-buffered saline (PBS) through vortex, and after centrifugation, resuspended in 1 mL of 70% ethanol overnight at 4 °C. The cells were then centrifuged and resuspended in 1 mL of NS buffer (10 mM Tris–HCl [pH 7.6], 250 mM sucrose, 1 mM EDTA [pH 8.0], 1 mM MgCl_2_, 0.1 mM CaCl_2_, 0.1 mM ZnCl_2_, 0.4 mM phenylmethylsulfonyl fluoride, 7 mM beta-mercaptoethanol). After centrifugation, the cells were resuspended and stained in propidium iodide (Fluka BioChemika) (0.5 mg/mL) in 0.2 mL of NS buffer containing 0.02 mL RNase A (1 mg/mL), followed by incubation at 4 °C overnight with agitation. The stained cells were then diluted into 1 mL of 50 mM Tris–HCl (pH 8.0) and sonicated for 13 s using a Fisher Scientific Sonic Dismembrator Model 100. Flow cytometry was performed with 10,000 cells on a Becton–Dickinson LSR II and analyzed using the ModFit LT™ (version 5.0, Verity Software House). Strains JEC20 and CDC15 were used as haploid references, while D14 was used as a diploid reference.

## Figures and Tables

**Table 1:** Genetic markers, genotypes, and fluconazole minimum inhibitory concentration (MIC) of mutation accumulation lines after 40 serial transfers (120 days).

Marker	Ch.	Y1	Y2	Y3	Y4	Y5	Y6	Y7	Y8	Y9	Y10	Ho	F1	F2	F3	F4	F5	F6	F7	F8	F9	F10	Ho
CNA00290	1	AD	AD	AD	AD	AD	AD	AD	AD	AD	A	0.9	A	AD	A	A	A	A	A	A	A	A	0.1
CNA07470	1	AD	AD	AD	AD	AD	AD	AD	AD	AD	A	0.9	A	AD	A	A	A	A	A	A	A	A	0.1
CNB00360	2	AD	AD	AD	D	AD	D	AD	AD	AD	AD	0.8	AD	AD	AD	AD	AD	AD	AD	AD	AD	AD	1
CNB03520	2	AD	AD	AD	AD	AD	AD	AD	AD	A	AD	0.9	AD	AD	AD	AD	AD	AD	AD	AD	AD	AD	1
CNB05710	2	AD	AD	AD	AD	AD	AD	AD	AD	A	AD	0.9	AD	AD	AD	AD	AD	AD	AD	AD	AD	AD	1
CNC00670	3	AD	AD	D	AD	AD	AD	AD	AD	AD	AD	0.9	AD	AD	AD	AD	AD	AD	AD	AD	AD	AD	1
CNC06110	3	AD	AD	AD	AD	AD	AD	AD	AD	AD	AD	1	AD	AD	AD	AD	AD	AD	AD	AD	AD	AD	1
CNC07810	3	AD	AD	AD	D	D	AD	AD	AD	AD	A	0.7	AD	AD	AD	AD	AD	AD	AD	AD	AD	AD	1
CND06160	4	AD	AD	AD	AD	AD	AD	AD	AD	AD	AD	1	AD	AD	AD	AD	AD	AD	AD	AD	AD	AD	1
RUM1	4	AD	AD	AD	AD	AD	AD	AD	AD	AD	AD	1	AD	AD	AD	AD	AD	AD	AD	AD	AD	AD	1
CNE01630	5	AD	AD	AD	AD	AD	AD	AD	AD	AD	AD	1	AD	AD	AD	AD	AD	AD	AD	AD	AD	AD	1
CNE03010	5	AD	AD	AD	AD	AD	AD	AD	AD	AD	AD	1	AD	AD	AD	AD	AD	AD	AD	AD	AD	AD	1
CNE05150	5	AD	AD	AD	AD	AD	AD	AD	AD	AD	AD	1	AD	AD	AD	AD	D	AD	AD	AD	AD	AD	0.9
CNF00290	6	AD	AD	AD	AD	AD	AD	AD	AD	AD	AD	1	AD	AD	AD	AD	AD	AD	AD	AD	AD	AD	1
CNF02400	6	AD	AD	AD	AD	AD	AD	AD	AD	AD	AD	1	AD	AD	AD	AD	AD	AD	AD	AD	AD	AD	1
CNF04800	6	AD	AD	AD	AD	AD	AD	AD	AD	AD	AD	1	AD	AD	AD	AD	AD	AD	AD	AD	AD	AD	1
CNG01240	7	AD	AD	AD	AD	AD	AD	AD	AD	AD	AD	1	AD	AD	AD	AD	AD	AD	AD	AD	AD	AD	1
CNG03250	7	AD	AD	AD	AD	AD	AD	AD	AD	AD	AD	1	AD	AD	AD	AD	AD	AD	AD	AD	AD	AD	1
CNG04610	7	AD	D	AD	AD	AD	AD	AD	AD	AD	AD	0.9	AD	AD	AD	AD	AD	AD	AD	AD	AD	AD	1
CNH00030	8	AD	AD	AD	AD	AD	AD	AD	AD	AD	AD	1	AD	AD	AD	AD	AD	D	AD	AD	AD	AD	0.9
CNH03360	8	AD	AD	AD	AD	AD	AD	AD	AD	AD	AD	1	AD	AD	AD	AD	AD	AD	AD	AD	AD	AD	1
CNI00070	9	AD	AD	AD	AD	AD	AD	AD	AD	AD	AD	1	AD	AD	AD	AD	AD	AD	AD	AD	AD	A	0.9
CNI04370	9	AD	AD	AD	AD	AD	AD	AD	AD	AD	AD	1	AD	AD	AD	AD	AD	AD	AD	AD	AD	A	0.9
CNJ00070	10	AD	AD	AD	AD	AD	AD	AD	AD	AD	AD	1	AD	AD	AD	AD	AD	AD	AD	AD	AD	AD	1
CNJ02920	10	AD	AD	AD	AD	AD	AD	AD	AD	AD	AD	1	AD	AD	AD	AD	AD	AD	AD	AD	AD	AD	1
CNK00170	11	AD	AD	AD	AD	AD	AD	AD	AD	AD	AD	1	AD	AD	AD	AD	AD	AD	AD	AD	AD	AD	1
CNK03410	11	AD	AD	AD	AD	AD	AD	AD	AD	AD	AD	1	AD	AD	AD	AD	AD	AD	AD	AD	AD	AD	1
CNL04620	12	AD	AD	AD	AD	AD	AD	AD	AD	AD	D	0.9	AD	AD	AD	AD	AD	AD	AD	AD	AD	AD	1
CNL06810	12	AD	AD	AD	AD	AD	AD	AD	AD	AD	AD	1	AD	A	AD	AD	AD	AD	AD	AD	AD	AD	0.9
CNM00630	13	AD	AD	AD	AD	AD	A	AD	AD	AD	AD	0.9	AD	AD	AD	D	AD	AD	AD	AD	AD	AD	0.9
CNM02560	13	AD	AD	AD	AD	AD	A	AD	AD	AD	AD	0.9	AD	AD	AD	D	AD	AD	AD	AD	AD	AD	0.9
CNN0060	14	AD	A	AD	AD	AD	AD	AD	AD	AD	A	0.8	AD	A	AD	AD	A	AD	AD	AD	AD	AD	0.8
CNN02060	14	AD	A	AD	AD	AD	AD	AD	AD	AD	AD	0.9	AD	A	AD	AD	A	A	AD	AD	AD	AD	0.7
Ho		1	0.9	1	1	1	0.9	1	1	0.9	0.8	1	0.9	0.9	0.9	0.9	0.9	0.9	0.9	0.9	0.9	0.9	0.9
MIC		32	32	32	16	32	16	32	32	32	128		128	>128	>128	>128	>128	>128	>128	>128	128	>128	

Note: Y-series were lines maintained on YEPD medium without fluconazole. F-series lines were maintained on YEPD medium supplemented with 64 µg/mL of fluconazole. Ch: Chromosome number where markers are located on the JEC21 genome; A: Allele from the C. *neoformans* strain CDC15; D: Allele from the C. *deneoformans* strain JEC20; AD: Alleles from both CDC15 and JEC20; Ho: Observed heterozygosity; MIC: Minimum inhibitory concentration of fluconazole (in µg/mL) based on microbroth dilution method. Highlighted in yellow are homozygous.

**Table 2:** Genotypes of the Y10 series and the 10 F-series mutation accumulation lines at the two marker loci CNA00290 and CNA07470 on Chromosome 1 over the six-time points.

	Y10	F1	F2	F3	F4	F5	F6	F7	F8	F9	F10
Day 0	AD	AD	AD	AD	AD	AD	AD	AD	AD	AD	AD
Day 15	AD	A	AD	A	A	AD	AD	A	AD	AD	A
Day 30	AD	A	AD	A	A	AD	AD	A	A	A	A
Day 45	A	A	AD	A	A	A	AD	A	A	A	A
Day 60	A	A	AD	A	A	A	A	A	A	A	A
Day 75	A	A	AD	A	A	A	A	A	A	A	A
Day 120	A	A	AD	A	A	A	A	A	A	A	A

A: Allele from the C. *neoformans* strain CDC15; D: Allele from the C. *deneoformans* strain JEC20; AD Alleles from both CDC15 and JEC20.

## Supplementary Materials

The following are available online at https://www.mdpi.com/2076-0817/9/1/3/s1, Figure S1: Colony Size (± standard deviation) for Each of the 20 Mutation Accumulation Lines.
