# Supplementary material for: Genetic Changes in Experimental Populations of a Hybrid in the Cryptococcus neoformans Species Complex
Source: Pathogens. 2019 Dec 18;9(1):3. doi: 10.3390/pathogens9010003 (PMC7168662; doi:10.3390/pathogens9010003)
Supplement: Supplementary file 1 [file pathogens-09-00003-s001.pdf]

Supplementary Figure S1. Colony size (± standard deviation) for each of the 20 mutation accumulation lines. Figure S1A shows the ten lines evolved on YEPD agar without fluconazole. Figure S1B shows the ten lines evolved on YEPD agar with 64µg/ml of fluconazole.

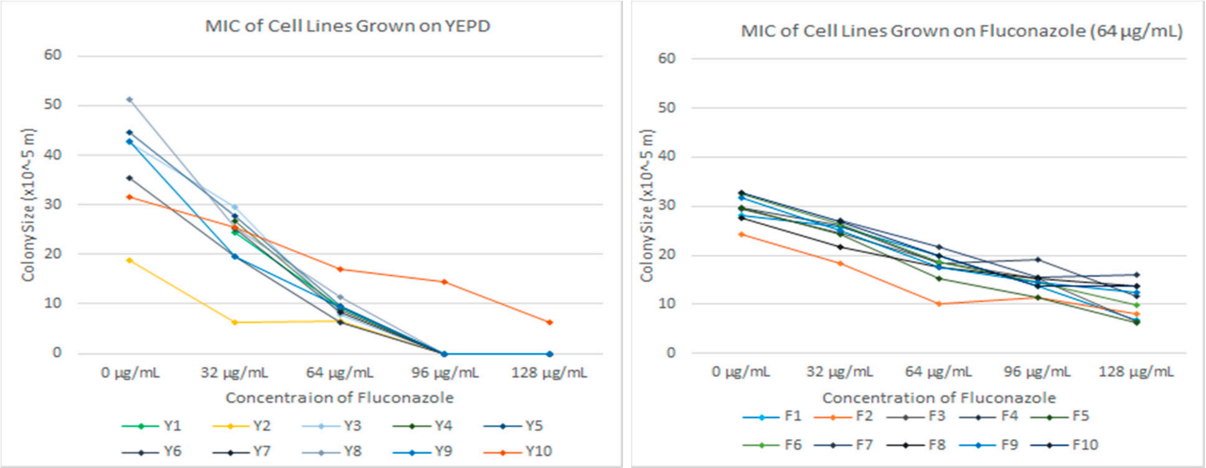

(**A**) (**B**)
